# The Relationship Between Crime-Related Television Viewing and Perceptions of the Death Penalty: Results of a Large Cross-Sectional Survey Study

**DOI:** 10.3389/fpsyg.2021.715657

**Published:** 2021-07-21

**Authors:** Benedikt Till, Florian Arendt, Thomas Niederkrotenthaler

**Affiliations:** ^1^Unit Suicide Research & Mental Health Promotion, Department of Social and Preventive Medicine, Center for Public Health, Medical University of Vienna, Vienna, Austria; ^2^Wiener Werkstaette for Suicide Research, Vienna, Austria; ^3^Department of Communication, University of Vienna, Vienna, Austria

**Keywords:** death penalty, television, cultivation theory, crime shows, perceptions of reality

## Abstract

**Background:** According to cultivation theory, distorted representations of social reality on television can lead to distorted perceptions of reality among viewers. For example, the death penalty has been abolished in most Western countries a long time ago, but is often portrayed or mentioned in US crime shows, which are very popular outside the United States. Previous research suggests that the amount of television viewing can be associated with erroneous perceptions of the use of the death penalty—even when the death penalty is not used in the respective country. Unfortunately, available evidence on this association is inconclusive.

**Methods:** In a cross-sectional web-based survey, we used quota-based sampling techniques to recruit 1,002 individuals representative of the Austrian population in terms of gender, age, education, and region of residence. We asked about their weekly amount of television viewing and use of US crime dramas and measured their beliefs on the use of the death penalty in Austria.

**Results:** Although television viewing in general was not associated with erroneous perceptions of the death penalty (i.e., no overall across-the-board cultivation effect), data analysis provided supporting evidence for the idea of a genre-specific cultivation effect: The more US crime shows participants watched, the more likely they were to mistakenly believe that the death penalty is used in Austria. This association held true even after controlling for the gender, age, and education of participants.

**Conclusion:** The finding that watching US crime shows, which are based on social reality in the United States, is associated with Austrian viewers' confusion with regards to perceptions of the death penalty is consistent with the genre-specific cultivation hypothesis. Some viewers may be guided more by mediated reality than by actual social reality.

## Introduction

One of the most important and debated theories for understanding the social and cultural impacts of media has been cultivation theory (Morgan et al., 2015), which focuses on the question whether television contributes to viewers' conceptions of social reality (Gerbner and Gross, 1976; Gerbner et al., 1986). In fact, the basic cultivation hypothesis predicts that the more individuals watch television and thus “live in the television world,” the more they show perceptions of social reality consistent with the “television world” (Gerbner and Gross, 1976; Gerbner et al., 1986; Morgan and Shanahan, 2010; Morgan et al., 2015). Television, as has been argued extensively, has replaced reading and interpersonal narrative as the primary means of storytelling for many citizens and thus has become the source of the most broadly shared images and messages in history (Gerbner and Gross, 1976; Gerbner et al., 1986). Based on this idea, it is believed that if television represents a constant flow of remarkably consistent depictions about a specific aspect of social reality, cumulative exposure to these messages may subtly influence audiences' perceptions of these aspects of social reality (Gerbner and Gross, 1976; Gerbner et al., 1986; Morgan and Shanahan, 2010; Morgan et al., 2015).

The problem in those perceptions is that content-analytic research suggests that many parts of the world of television differ in important and sometimes dramatic ways from the “real” world (Shrum and Lee, 2012; Shrum, 2017). Many aspects of the world portrayed in fictional television are exaggerated or incorrect. For example, reviews of cultivation research of the last five decades have shown that the world presented on television tends to be more affluent, but also more violent, dangerous, and untrustworthy (Gerbner and Gross, 1976; Gerbner et al., 1986; Morgan and Shanahan, 2010; Shrum and Lee, 2012; Shrum, 2017). Also, the prevalence of certain occupations or population groups are portrayed disproportionally more frequently than others (e.g., police officers, lawyers, doctors, young people, criminals) (Gerbner and Gross, 1976; Gerbner et al., 1986; Shrum and Lee, 2012; Shrum, 2017). As a result, those who spend more time watching television are, according to the basic cultivation hypothesis, more likely to make judgments about the world that more closely reflect mediated reality presented on television (Gerbner and Gross, 1976; Gerbner et al., 1986; Morgan and Shanahan, 2010; Shrum and Lee, 2012; Morgan et al., 2015; Shrum, 2017), including the overestimation of certain occupations in the population and the adaption of certain beliefs and attitudes about society (e.g., fear of crime due to the overestimation of crimes) (Gerbner and Gross, 1976; Gerbner et al., 1986; Morgan and Shanahan, 2010).

One of the main cognitive mechanisms underlying cultivation effects is heuristic processing (Shrum and Lee, 2012; Shrum, 2017). The basic idea is that television viewing increases the accessibility of concepts often portrayed on television in viewers' minds (i.e., the ease with which specific examples come to their mind) (Tversky and Kahneman, 1973). For example, by regularly watching television shows that feature doctors, the accessibility of the concept “doctor” is assumed to increase in viewers' minds, which may result in an overestimation of the number of individuals in a society working as doctors. Such effects may influence individuals' judgments in a subsequent situation. In fact, when people construct such judgments about the world, they do not carefully consider all information in their memory, but use mental shortcuts and rely on mental content that is easy to recall (i.e., high accessibility), including much information from television for heavy viewers—a type of mental processing termed heuristic processing (Shrum and Lee, 2012; Shrum, 2017). Importantly in this regard is what some scholars have described as ‘source confusion' (Mares, 1996): People may memorize an information over a longer time, but they may forget the source of the information. In other words, people may forget where they originally learned something, which may result in fiction to be misconstrued as fact (Mares, 1996). Consistent with this idea, findings from psychological research on source monitoring, which refers to processes involved in making attributions about the origins of memories, knowledge, and beliefs (Johnson et al., 1993), indicate that people do not automatically bind source information with their memories (Ünal et al., 2016). As a result, this can lead to inaccurate source attributions or source memory errors (Johnson, 1997; Ünal et al., 2016). For example, individuals tend to mistakenly attribute self-generated representations to perceptions or add knowledge, expectations, and other inferences to actual events (Johnson, 1997; Goldstein, 2015; Ünal et al., 2016). Importantly for the current study, people may also use information from television, including fictional programs, as a source about events, occupational groups, or other concepts that are not experienced directly, first-hand (Surette, 2007).

Whereas “classic” cultivation research, which started in the 1960s, was concerned with the effects of television in general, the new media environment with today's large variety of cable programs and the spread of internet-connected television and streaming services has increasingly turned media researchers' interests toward “genre-specific cultivation” in the last two decades (Grabe and Drew, 2007; Bilandzic and Busselle, 2008; Lee and Niederdeppe, 2010). The central idea is that there is not one single, uniform “television world” with a single meta-narrative, which may have been the case many decades ago when the number of available television channels was relatively low. Many scholars have argued that there are more “television worlds” in current days and thus different genres (e.g., romantic comedy, crime, etc.) may represent and cultivate different versions of specific aspects of social reality (Grabe and Drew, 2007; Bilandzic and Busselle, 2008; Lee and Niederdeppe, 2010).

## Television Viewing and Perceptions of the Death Penalty

Since its original conception, research on cultivation theory has always had a strong focus on beliefs about the justice system and crime. For example, individuals with high television consumption have been found to overestimate the amount of violent crimes in society and the number of people working as police officers, have unrealistic expectations of the police, and tend to experience the world as a dangerous place (Gerbner and Gross, 1976; Gerbner et al., 1986; Perlmutter, 2000; Morgan and Shanahan, 2010). These associations have not only been found for the overall amount of television viewing, but also for specific television genres such as the crime genre (Morgan and Shanahan, 2010). For example, viewing local and national news, police reality shows, and fictional crime dramas has been found to be associated with fear of crime, low levels of trust in other people, handgun ownership, misconceptions about the justice system, and support of the death penalty (Eschholz et al., 2003; Holbert et al., 2004; Goidel et al., 2006; Salmi et al., 2007).

Based on this evidence, it comes as no surprise that media researchers in different countries have begun to explore whether cultivation may also be related to beliefs about basic principles of the justice system such as the death penalty. “Confusion” about core societal realities such as the existence of the death penalty in one's own country may have important social and political implications. For example, according to the Marshall hypothesis, most people support the death penalty due to a lack of knowledge about the death penalty and the justice system along with fear of crime (Mitchell, 2006). If television viewing (or watching US crime shows) is reflected in fundamental beliefs about core societal aspects (e.g., the justice system), then people may make decisions about their life (e.g., voting for a political party, buying a gun, supporting the death penalty, etc.) partly based on fictional facts (Holbert et al., 2004). Importantly, this phenomenon of confusion about core societal aspects may also transfer to other aspects of life such as beliefs or decisions related to romantic relationships, occupation, or financial aspects (Gerbner and Gross, 1976; Gerbner et al., 1986).

It is important to note that US-American crime shows are very popular and frequently watched in most Western countries (Oberman, 2017; European Audiovisual Observatory, 2019). In other words, television viewers around the world are exposed to a reality that may more accurately reflect certain aspects of the American justice system than the justice system in the respective other country. There is, however, one clear and striking distinction between the justice system in the United States and many other countries: The United States remain the only Western industrialized nation to continue to routinely sentence capital offenders to death and execute these sentences (Amnesty International, 2020). Thus, the death penalty is a reality of the justice system in the United States, but not, for example, in Canada or in any country in the EU.

## Available Empirical Evidence

In a sample of 322 respondents living in Austria, Till et al. (2016) asked five questions related to the death penalty in Austria (e.g., “How many inmates in Austria do you think are currently sitting on death row?” or “How many inmates in Austria do you think were executed by lethal injection over the past five years?”). The correct answer for all questions was “zero,” since the death penalty was abolished in Austria more than 50 years prior to this study (the last execution was March 24, 1950) (Leder, 1986; Sabitzer, 2010). Despite this, 11% of the respondents gave at least one incorrect answer (Till et al., 2016). The authors found that greater amount of weekly television exposure predicted a greater likelihood of having mistaken beliefs on whether the death penalty is used in Austria. This effect remained significant even when age and education were statistically controlled. Moreover, this association was not only found between television viewing and each individual death penalty question, but also with regards to the dichotomized belief in the death penalty sum score (i.e., any vs. no error among the five death penalty questions) (Till et al., 2016).

Till et al. (2016) explained this finding with cultivation theory and the high amount of US crime shows on Austrian television, which are obviously based on facts of the US justice system, with the death penalty being a central and very specific aspect of this system. Till et al. (2016) argued that, consistent with the above mentioned accessibility model proposed by Shrum (Shrum and Lee, 2012; Shrum, 2017), the more viewers watched US crime shows on television, the higher was the accessibility of the concept of the death penalty in viewers' minds. When confronted with questions related to the death penalty, respondents provided answers based on what was easily accessible to them (i.e., memory traces related to the death penalty) (Till et al., 2016).

The study by Till et al. (2016) was not the first that investigated associations between television viewing and perceptions of the death penalty. Truong (2011) conducted a very similar survey five years earlier with 173 undergraduate students in Canada. Respondents who believed that Canada practices the death penalty reported a significantly higher amount of television viewing than those who did not share this belief. This difference, however, became statistically non-significant once socio-demographic variables were taken into account.

In an attempt to replicate the original two studies from Canada and Austria (Truong, 2011; Till et al., 2016), Boch et al. (2019) surveyed a convenience sample of 597 participants living in Austria and found television viewing predicted only one of the five death penalty items, and there was no association with the dichotomized sum core (i.e., any vs. no error among the five death penalty questions). There was also no association between the amount of US crime show use and the dichotomized death penalty sum score (associations between watching US crime shows and the individual death penalty items were not reported). Despite reporting a small, but significant positive association between television viewing and perceptions of the death penalty in their preliminary meta-analytic evaluation of findings from their own and the two previous studies (Truong, 2011; Till et al., 2016; Boch et al., 2019), and despite cultivation research generally reporting small associations and effects (but still socially meaningful because of their cumulative nature) (Shrum and Lee, 2012; Morgan et al., 2015), Boch et al. (2019) argued that the correlative findings may be “a research artifact.” Boch et al. (2019) also highlighted a lack of assessment of preferences for US crime shows and information related to less traditional forms of television viewing (e.g., online streaming or DVDs) as short-comings of previous research, and they recommended using a traditional sum score ranging from 0 to 5 to measure respondents' perceptions of the death penalty (instead of assessing each item individually or using a dichotomized sum score) for future research.

Due to the inconclusive evidence from previous studies (Truong, 2011; Till et al., 2016; Boch et al., 2019), the current study aimed to provide additional empirical evidence on associations between television viewing and perceptions of the death penalty. Most importantly, the current study used a large quota-based sample that is representative of the Austrian population in terms of gender, age, education, and region of residence. Previous studies only relied on convenience sampling techniques (i.e., samples consisting exclusively or predominantly of university students). We investigated associations of television viewing and watching US crime shows with perceptions of the death penalty in Austria as recommended by Boch et al. (2019). We hypothesized that the more respondents watch US crime shows (genre-specific cultivation hypothesis) and television in general (overall across-the-board cultivation hypothesis), the more likely they are to inaccurately assume that the death penalty is used in Austria.

## Materials and Methods

### Participants

We conducted a cross-sectional web-based survey based on quota-sampling techniques between December 11 and December 22, 2020. A marketing company, *Ipsos*, recruited a sample representative of the Austrian population of 16 years and older in terms of gender, age, education, and region of residence from an online panel consisting of 30,000 registered members. Based on population statistics from Statistics Austria, which is Austria's official institution for statistics research, a quota sampling methodology was employed with quotas based on gender (i.e., male, female, diverse), age (i.e., 16–29, 30–39, 40–49, 50–59, 60–69, 70+), region (i.e., all nine federal states of Austria), and education (i.e., compulsory, secondary, university education). Furthermore, we weighted the dataset across combinations of all four variables by using the random iterative method via *Rim Weighting Excel Macro* (Survey Science, 2016) to achieve representativeness across combinations of all four variables by matching the respective joint distributions, which further improves the precision of the survey estimates.

Participants were invited by emails, which were sent out by *Ipsos* based on the open quotas and individual response probabilities estimated by the panel algorithm. In total, 7,159 invitations were sent to panel members and 1,546 individuals accepted the invitation and started the survey. Whereas 375 respondents were screened out because quotas were already full and 169 respondents dropped out before completing the survey, 1,002 participants completed the survey. Due to the low number of observed frequencies, one participant who indicated “other gender” (*n* = 1) was excluded from the statistical analysis, resulting in a total of *n* = 1,001 included participants. Respondents who completed the entire survey were compensated by *Ipsos* for their time. According to company policy, respondents receive 1.00–2.50 Euro (depending on the length of the respective survey) and one raffle ticket for each completed survey.

### Measures

#### Watching US Crime Shows

Consistent with Boch et al. (2019), the weekly amount of watched US crime shows was assessed using two questions with an open-response format. Respondents were asked to report the average number of hours of US crime shows (including DVDs, online streaming, and over-the-top media platforms) they watch on a typical weekday (Monday to Thursday) and on a typical weekend days (Friday to Sunday). Entries above 24 h a day were not possible. Based on these entries, the total number of hours per week spend on watching US crime shows was calculated for each respondent.

#### Television Viewing

Consistent with previous research (Truong, 2011; Till et al., 2016; Boch et al., 2019), weekly television viewing was assessed using two questions with an open-response format. Respondents were asked to report the average number of hours of television (including DVDs, online streaming, and over-the-top media platforms) they watch on a typical weekday (Monday to Thursday) and on a typical weekend days (Friday to Sunday). Entries above 24 h a day were not possible. Based on these entries, the total number of hours per week spend on watching television was calculated for each respondent.

#### Perceptions of the Death Penalty

To assess participants' perceptions of the death penalty in Austria, we used five questions measuring participants' beliefs and knowledge of the use of the death penalty in Austria. These questions were originally developed by Truong (2011) and adapted for Austria by Till et al. (2016). The participants were asked to answer the following five open-ended questions: (1) How many inmates in Austria do you think are currently sitting on death row (i.e., the number of inmates awaiting execution)?; (2) How many inmates in Austria do you think were executed by lethal injection over the past 5 years (2016 to 2020)?; (3) How many inmates in Austria do you think were executed by lethal injection over the past 25 years (1996 to 2020)?; (4) How many inmates in Austria do you think were executed by electric chair over the past 5 years (2016 to 2020)?; and (5) How many inmates in Austria do you think were executed by electric chair over the past 25 years (1996 to 2020)? Each question was followed by the instruction “Please just type the number, e.g., ‘6' ” in brackets. Since the death penalty was officially abolished in Austria in 1968 and not executed since 1950 (Leder, 1986; Sabitzer, 2010), the correct answer for all questions was “0.” As a result, all answers indicating the number “0” were coded as correct, whereas all other entries were coded as incorrect. Consistent with the original study (Till et al., 2016), we calculated a dichotomized total score indicating whether a respondent answered all five questions related to the death penalty correctly or gave at least one incorrect answer. Thus, the total score was coded “0,” if all five questions were answered correctly, and coded “1,” if any of the questions were answered incorrectly. In addition, based on the recommendation of Boch et al. (2019), we also calculated a traditional sum score across all five questions with the score ranging between 0 and 5.

### Data Analysis

We calculated six binary logistic regression analyses using the entry method to estimate associations between television viewing and perceptions of the death penalty. For each binary logistic regression analysis, one of the answers to the five individual questions on the death penalty or the dichotomized total score were used as dependent variable. Weekly use of television and US crime shows served as predictors. Furthermore, we conducted one multiple linear regression analysis with television and US crime show use as predictors and the traditional sum score for perceptions of the death penalty (score range: 0–5) as dependent variable. All regression analyses controlled for participant gender, age, and education (i.e., with one dummy variable for individuals with high school degree and one dummy variable for individuals with college degree as highest completed level of education; reference category = no high school). An inter-correlation matrix with Pearson correlations was used to calculate bivariate associations between all variables. All statistical analyses were conducted with both, weighted and unweighted data, respectively.

We performed a sensitivity analysis to assess whether patterns differed if unweighted instead of weighted data were used. Tables providing results of descriptive statistics, bivariate Pearson correlations, and regression analyses with unweighted data can be found in Supplementary Tables 1–4. Results for analyses with unweighted data were very similar to those with weighted data. Furthermore, we performed a sensitivity analysis to test whether patterns differed if extreme outliers (i.e., participants who provided possible, but unrealistically high entries) for television viewing or US crime show use were removed. For this analysis, 31 participants who provided entries above three standard deviations of the respective mean of television viewing or US crime show use were excluded (Pukelsheim, 1994). Results for analyses with and without these extreme outliers were almost identical (data available upon request).

## Results

### Descriptive Statistics

Of the 1,001 included respondents, 516 (51.6%) were women and 485 (48.4%) were men. In terms of age group, 207 (20.7%) were 16–29, 156 (15.6%) were 30–39, 196 (19.6%) were 40–49, 162 (16.2%) were 50–59, 125 (12.5%) were 60–69, and 154 (15.4%) were 70+ years old. In terms of highest completed education, 746 (74.5%) had an education level below high school, 137 (13.7%) had a high school degree, and 118 (11.8%) had college or university education. The average duration of weekly television use was 28.9 h (*SD* = 20.8) and the average duration of weekly US crime show use was 8.3 h (*SD* = 13.0). A total of 180 respondents (18.0%) answered at least one of the five death penalty questions incorrectly. See Table 1 for an overview of the answers provided for each of the death penalty questions along with the descriptive statistics for television and US crime show use. Younger individuals had significantly more inaccurate beliefs about the death penalty in Austria than older individuals across all items. Individuals with a college degree had a higher probability of mistakenly believing that capital offenders in Austria were executed by lethal injection or electric chair in the past five years and scored higher on the traditional sum score. Furthermore, women tended to be more likely to mistakenly believing that capital offenders in Austria were executed by lethal injection or electric chair in the past 25 years and scored higher on the dichotomized sum score. Table 2 provides a matrix of bivariate correlations between all variables included in the regression analyses. See Table 3 and Table 4 for an overview of the results of the regression analyses.

**Table 1:** Descriptive statistics of perceptions of the death penalty and television viewing among study participants (*n* = 1001).

**Question**	**Answer**	***N***	**%**	**TV viewing**		**Watching US crime shows**	
				***M***	***SD***	***M***	***SD***
Anyone on death row	Correct	864	86.3	28.61	20.19	7.33	11.34
	Incorrect	137	13.7	31.06	24.05	14.70	19.63
Lethal injection past 5yr	Correct	896	89.5	28.84	20.68	7.61	12.50
	Incorrect	105	10.5	29.83	21.55	14.55	15.57
Lethal injection past 25yr	Correct	854	85.4	28.81	20.19	7.57	11.51
	Incorrect	147	14.6	29.69	23.92	12.83	19.13
Electric chair past 5yr	Correct	917	91.6	28.87	20.57	7.63	12.50
	Incorrect	84	8.4	29.78	22.86	16.13	15.89
Electric chair past 25yr	Correct	883	88.2	28.97	20.73	7.65	12.56
	Incorrect	118	11.8	28.71	21.12	13.49	15.21
All five questions combined^a^	Correct	821	82.0	28.85	20.22	7.43	11.47
	Incorrect	180	18.0	29.35	23.12	12.47	17.99

*Values are weighted absolute (N) and relative (%) frequencies of participants answering the five questions on the death penalty correctly or incorrectly as well as their means (M) and standard deviations (SD) in terms of weekly television consumption and weekly consumption of US crime shows*.

a *This parameter was coded as correct, if all five questions on the death penalty were answered correctly, and as incorrect, if any of the five questions were answered incorrectly*.

**Table 2:** Correlation matrix: pearson correlations between perceptions of the death penalty, television viewing, gender, age, and education.

	**X1**	**X2**	**Y1**	**Y2**	**Y3**	**Y4**	**Z1**	**Z2**	**Z3**	**Z4**	**Z5**	**Z6**	**Z7**
Television viewing (X1)	1.00												
Watching US crime shows (X2)	0.41^***^	1.00											
Gender (Y1)^a^	−0.01	−0.06*	1.00										
Age (Y2)	0.19^***^	−0.03	−0.21^***^	1.00									
Education: High school (Y3)	−0.05	−0.03	−0.04	−0.03	1.00								
Education: College (Y4)	−0.08^*^	−0.02	−0.06	0.05	−0.15^***^	1.00							
Anyone on death row (Z1)	0.04	0.19^***^	0.07^*^	−0.25^***^	0.00	0.04	1.00						
Lethal injection past 5yr (Z2)	0.02	0.16^***^	0.06^*^	−0.26^***^	−0.01	0.07^*^	0.77^***^	1.00					
Lethal injection past 25yr (Z3)	0.02	0.14^***^	0.11^***^	−0.30^***^	−0.01	0.03	0.70^***^	0.81^***^	1.00				
Electric chair past 5yr (Z4)	0.01	0.18^***^	0.04	−0.20^***^	−0.01	0.08^*^	0.70^***^	0.86^***^	0.70^***^	1.00			
Electric chair past 25yr (Z5)	0.00	0.14^***^	0.11^***^	−0.27^***^	0.03	0.03	0.68^***^	0.81^***^	0.85^***^	0.81^***^	1.00		
All 5 questions combined^b^ (Z6)	0.01	0.15^***^	0.11^***^	−0.33^***^	−0.01	0.01	0.85^***^	0.73^***^	0.88^***^	0.64^***^	0.78^***^	1.00	
Death penalty sum score^c^(Z7)	0.02	0.18^***^	0.09^**^	−0.29^***^	0.00	0.05	0.86^***^	0.94^***^	0.91^***^	0.89^***^	0.92^***^	0.87^***^	1.00

*Values are correlation coefficients (r) from weighted Pearson correlations;*

* *p < 0.05*,

** *p < 0.01*,

*** *p < 0.001 (two-tailed)*.

a *Reference group: Male*.

b *This parameter was coded as correct, if all five questions on the death penalty were answered correctly, and as incorrect, if any of the five questions were answered incorrectly*.

c *This parameter is a sum score based on the responses to the five death penalty questions (0 = correct answer, 1 = incorrect answer) ranging from 0 to 5*.

**Table 3:** Results of binary logistic regression analyses to predict perceptions of the death penalty among study participants.

**Question**		**TV viewing**	**Watching US** **crime shows**	**Gender^**a**^**	**Age**	**Education:** **High school**	**Education:** **College**
Anyone on death row *χ^2^*(6) = 101.73, *p* <0.001 Nagelkerkes *R^2^* =0.18	*b* (SE) Wald OR (95% CI)	0.00 (0.01) 0.04 1.001 (0.991−1.012)	0.04 (0.01) 24.43^***^ 1.038 (1.023−1.053)	0.25 (0.20) 1.47 1.279 (0.860−1.902)	−0.51 (0.07) 50.29^***^ 0.599 (0.520−0.690)	−0.10 (0.30) 0.10 0.910 (0.510−1.624)	0.48 (0.29) 2.76 1.616 (0.918−2.846)
Lethal injection past 5yr *χ^2^*(6) = 103.55, *p* <0.001, Nagelkerkes *R^2^* =0.20	*b* (SE) Wald OR (95% CI)	0.00 (0.01) 0.10 0.998 (0.986−1.010)	0.04 (0.01) 18.31^***^ 1.036 (1.019−1.053)	0.22 (0.23) 0.90 1.242 (0.794−1.943)	−0.65 (0.09) 53.02^***^ 0.522 (0.439−0.622)	−0.28 (0.34) 0.69 0.753 (0.386−1.472)	0.71 (0.31) 5.40^*^ 2.042 (1.119−3.728)
Lethal injection past 25yr *χ^2^*(6) = 131.48, *p* <0.001, Nagelkerkes *R^2^* =0.22	*b* (SE) Wald OR (95% CI)	0.00 (0.01) 0.09 1.002 (0.991−1.012)	0.03 (0.01) 14.72^***^ 1.029 (1.014−1.045)	0.46 (0.20) 5.13* 1.582 (1.064−2.354)	−0.65 (0.08) 72.37^***^ 0.521 (0.449−0.606)	−0.29 (0.30) 0.99 0.746 (0.418−1.329)	0.40 (0.29) 1.85 1.487 (0.839−2.635)
Electric chair past 5yr *χ^2^*(6) = 79.45, *p* <0.001, Nagelkerkes *R^2^* =0.18	*b* (SE) Wald OR (95% CI)	−0.01 (0.01) 0.93 0.993 (0.980−1.007)	0.04 (0.01) 22.42^***^ 1.042 (1.024−1.060)	0.17 (0.25) 0.46 1.183 (0.726−1.928)	−0.55 (0.09) 34.40^***^ 0.579 (0.482−0.695)	−0.26 (0.38) 0.47 0.770 (0.363−1.631)	0.84 (0.32) 6.84^**^ 2.316 (1.234−4.344)
Electric chair past 25yr *χ^2^*(6) = 106.76, *p* <0.001, Nagelkerkes *R^2^* =0.20	*b* (SE) Wald OR (95% CI)	0.00 (0.01) 0.49 0.996 (0.984−1.008)	0.03 (0.01) 17.59^***^ 1.034 (1.018−1.051)	0.49 (0.22) 4.91^*^ 1.634 (1.058−2.522)	−0.61 (0.08) 53.47^***^ 0.545 (0.463−0.641)	0.05 (0.30) 0.03 1.056 (0.588−1.896)	0.40 (0.32) 1.61 1.491 (0.804−2.766)
All five questions combined^b^ *χ^2^*(6) = 145.38, *p* <0.001, Nagelkerkes *R^2^* =0.22	*b* (SE) Wald OR (95% CI)	0.00 (0.01) 0.03 1.001 (0.991−1.011)	0.03 (0.01) 16.83^***^ 1.030 (1.015−1.044)	0.36 (0.19) 3.85^*^ 1.438 (1.000−2.067)	−0.63 (0.07) 83.71^***^ 0.534 (0.467−0.611)	−0.30 (0.27) 1.18 0.744 (0.436−1.270)	0.23 (0.28) 0.69 1.260 (0.730−2.175)

*Values are weighted unstandardized regression coefficients (b) with standard errors (SE) given in parentheses, Wald statistics, and odds ratios (OR) with 95% confidence intervals (95% CI) given in parentheses;*

* *p < 0.05*,

** *p < 0.01*,

*** *p < 0.001 (two-tailed)*.

a *Reference group: Male*.

b *This parameter was coded as correct, if all five questions on the death penalty were answered correctly, and as incorrect, if any of the five questions were answered incorrectly*.

**Table 4:** Results of multiple linear regression analysis to predict perceptions of the death penalty^a^ among study participants.

**Predictor**	***R^**2**^***	**Δ*F***	***B***	**SE *B***	**β**	***t***
	0.12	22.37^***^				
TV viewing			0.00	0.00	0.00	0.11
Watching US crime shows			0.02	0.00	0.18	5.36^***^
Gender^b^			0.14	0.09	0.05	1.54
Age			−0.23	0.03	−0.28	−8.74^***^
Education: High school			0.03	0.18	0.01	0.26
Education: College			0.32	0.14	0.07	2.34^*^

*Values are unstandardized (B) und standardized (β) regression coefficients, standard errors of the unstandardized regression coefficients (SE B), and t values (t) based on weighted data. Also reported are R^2^ and change in F value (ΔF) of the regression model;*

* *p < 0.05,*

** *p < 0.01,*

*** *p < 0.001 (two-tailed)*.

a *This parameter is a sum score based on the responses to the five death penalty questions (0 = correct answer, 1 = incorrect answer) ranging from 0 to 5*.

b *Reference group: Male*.

### Associations Between Television Viewing and Perceptions of the Death Penalty

Multivariate analyses revealed that watching US crime shows predicted answers to all five questions on the death penalty along with the dichotomized total score (see Table 3). The more hours of US crime shows participants reported viewing per week, the higher was the probability of an incorrect answer, i.e., mistakenly believing that the death penalty is used in Austria. Similarly, the results of the multiple linear regression analysis with the traditional sum score for perceptions of the death penalty as dependent variable indicated that watching US crime shows predicted perceptions of the death penalty (see Table 4). The more hours of US crime shows participants reported watching per week, the more perceptions on the death penalty were inaccurate. Thus, a positive association between watching US crime shows and (inaccurate) perceptions of the death penalty was found for all dependent variable scores (i.e., individual items, dichotomized sum score, and traditional sum score). This means that regardless of how the perceptions of the death penalty were operationalized/calculated, inaccurate beliefs were predicted by the amount of US crime show use. These associations were found to be robust, independent of individual differences in gender, age, and education, which were controlled for in all regression analyses. This pattern is consistent with the genre-specific cultivation hypothesis. Conversely, the amount of television viewing was not associated with perceptions of the death penalty, regardless of the score used as dependent variable (i.e., individual items, dichotomized sum score, and traditional sum score). Thus, the analysis did not provide supporting evidence for an overall across-the-board cultivation effect.

## Discussion

The current study aimed to provide additional empirical evidence on the association between amount of (US-based crime-related) television viewing and inaccurate beliefs about the death penalty (Truong, 2011; Till et al., 2016; Boch et al., 2019) and account for some limitations of previous studies already outlined in the literature (Boch et al., 2019). The results showed that the amount of US crime shows Austrians watched per week predicted a greater likelihood of endorsing misbeliefs on whether the death penalty is used within the Austrian justice system. In other words, the more US crime shows respondents watched (and thus the more Austrians “lived in the US televised social reality”), the greater was their “confusion” about the domestic use of the death penalty in Austria. This held true even when gender, age, and education were controlled in the statistical analysis.

The findings of the current study, which is the first in this line of research that used quota-based sampling techniques and was therefore representative of the Austrian population in terms of gender, age, education, and region of residence, are partly consistent with evidence from the original two studies in Austria and Canada (Truong, 2011; Till et al., 2016). In these two studies, there was a bivariate association between television viewing and misconceptions regarding the practice of the death penalty (in the Austrian study, this association also remained significant when controlled for age and education), and this link was explained with the high prevalence of US crime shows on Austrian and Canadian television (Truong, 2011; Till et al., 2016). In the current study, we have revised the measure of television viewing and added an additional measure for watching US crime shows based on previous recommendations (Boch et al., 2019). We found that watching US crime shows predicted confusion about the death penalty, which is consistent with a genre-specific cultivation effect (Grabe and Drew, 2007; Bilandzic and Busselle, 2008; Lee and Niederdeppe, 2010). However, in line with findings by Boch et al. (2019), there was no association with regards to television viewing in general. Therefore, the current study does not provide supporting evidence for an overall across-the-board cultivation effect.

The revision of the measures for television viewing in the current study based on recommendations by Boch et al. (2019) may have increased their precision and robustness compared to the previous studies. The explicit instruction for respondents to include the use of DVDs, online streaming, and over-the-top media platforms into their estimate for television viewing and US crime show use may explain the differences between the current work and previous studies (Truong, 2011; Till et al., 2016; Boch et al., 2019). Till et al. (2016) and Truong (2011) did not assess the use of DVDs, online streaming, and over-the-top media platforms, whereas Boch et al. (2019) assessed and analyzed it separately from television viewing. Our findings are consistent with the basic notion of genre-specific cultivation that individual beliefs and assumptions about the world are at least partly based on the most constant and recurring portrayals of social reality in the world of a television genre such as crime shows (Perlmutter, 2000; Eschholz et al., 2003; Holbert et al., 2004; Goidel et al., 2006; Grabe and Drew, 2007; Salmi et al., 2007; Bilandzic and Busselle, 2008; Lee and Niederdeppe, 2010; Till et al., 2016). The lack of association between television viewing in general and perceptions of the death penalty could be explained by the ever-increasing diversity of television programs. Other than US crime shows, there are only very few television programs in Austria that feature portrayals of the American justice system (and the death penalty). Consistent with this notion, exposure to specific television genres have often been found to be more robust predictors of cultivation than total television exposure (Potter, 2014).

It is also important to note that young age was the strongest predictor for inaccurate perceptions of the death penalty, which has also been observed in previous studies in Austria (Till et al., 2016; Boch et al., 2019). A possible explanation may be that young people have not experienced the changes in laws on the death penalty in Austria themselves in the 1950s and 1960s. As a result, older individuals may be more aware of the abolishment of the death penalty in Austria, whereas younger individuals may tend to rely more on (inaccurate) information resources such as television crime shows.

Another point that is important to discuss is the prevalence of confusion about the death penalty. Whereas 78% of participants of the study conducted in Canada gave at least one incorrect answer with regards to the death penalty (Truong, 2011), this rate was 18% in the current study and approximately 10% in the two previous studies in Austria (Till et al., 2016; Boch et al., 2019). This may suggest that this type of confusion is more prevalent in societies that are more geographically proximal to the United States or have a higher availability of American media. Furthermore, the current findings, which are based on a large quota-based sample, indicate that this type of confusion is more prevalent among television viewers in Austria than originally estimated (Till et al., 2016; Boch et al., 2019). The finding that watching US crime shows is associated with confusion about fundamental norms of society (i.e., a society's approval of the death penalty) is concerning, because this type of confusion may also be reflected in other areas of social reality (Gerbner and Gross, 1976; Gerbner et al., 1986; Eschholz et al., 2003; Holbert et al., 2004; Goidel et al., 2006; Salmi et al., 2007).

However, as sometimes mistakenly assumed by the media or other researchers (Boch et al., 2019), it is not likely that 18% of the Austrian population actually believes that the death penalty is used in Austria. As noted by Boch et al. (2019), if respondents are asked directly whether Austria currently practices the death penalty, the rate of incorrect answers is much lower. It is important to understand that our intention was to explore whether individuals, who tend to watch US crime shows, have “television facts” more accessible for recall, which may cause temporary confusion about core aspects of society when related cognitions are activated. People memorize information from television, including fictional programs such as crime shows, and in a subsequent situation (e.g., when generating a real word judgment), they provide spontaneous answers based on the most accessible information (Shrum and Lee, 2012; Shrum, 2017). This can lead to the emergence of contradicting information on some topics (e.g., the use of the death penalty), which may result in temporary confusion when they are recalled without specific context.

Improving media literacy in the general population may contribute to a reduction or even an elimination of undesired genre-specific cultivation effects. For example, several scholars have pointed out that some films and television programs provide a more realistic portrayal of social issues and can be used for educational purposes (Niemiec and Schulenberg, 2011; Russell and Waters, 2017). Previous research has shown that integrating popular media, including television, into school curricula helps students to think critically about media contents and question “television reality” and thus possibly help to dampen detrimental cultivation effects (Stevens, 2001). Furthermore, the lack of domestic television produced in Austria and other countries in the EU has been debated for many years (Der Standard, 2009). Despite some increases in the last few years, only 58% of fictional television in the EU are domestic productions (European Audiovisual Observatory, 2019). Greater investments in domestic television programs, which are more likely to portray events in a manner more consistent with the audience's social reality, may help to reduce the prevalence of US-television based misconceptions and myths.

### Strengths and Limitations

There are several strengths and limitations in this study. A strength was the use of a large quota-based sample that was representative of the Austrian population in terms of gender, age, education, and region of residence. Moreover, with an average of 4.13 h of daily television viewing time, television use was comparable to many other Western societies, including the United States (WorldAtlas, 2018; Nielsen, 2021). The current study has also some limitations. First, consistent with previous research (Truong, 2011; Till et al., 2016; Boch et al., 2019), we used a cross-sectional design for data collection, which allowed us to assess the prevalence and predictors of confusion about the death penalty in the Austrian population in a large population-based sample. However, conclusions with regard to causality could not be assessed with this study design. While it is possible that watching US crime shows has the potential to influence social perceptions of society such as the use of the death penalty, it is also possible that individuals with greater affinity toward US-American culture (and related death penalty beliefs) have a greater preference for US crime shows. Moreover, some unmeasured variables may also be responsible for the observed association, but were not assessed in our study. For example, we have controlled for education in our analyses, but not specifically for respondents' political education/knowledge or intelligence as defined and measured by standard intelligence tests used in psychological research (Sternberg and Ruzgis, 1994). We also did not control for how long an individual has already been living in Austria. However, the majority of immigrants in Austria come from countries in the EU or other countries that have also abolished the death penalty (Statistics Austria, 2021). Finally, the size of the reported associations was relatively small. However, effects and associations reported for cultivation-related research are, for the most part, generally small, but still meaningful because of their cumulative nature (Shrum and Lee, 2012; Morgan et al., 2015) and equivalent to the average measures of association of many findings in psychological research (Meyer et al., 2001; Hemphill, 2003; Richard et al., 2003).

## Conclusions

Cultivation research has repeatedly shown that peoples' beliefs about the world tend to reflect the amount of time people spend watching television or specific genres of television (Gerbner and Gross, 1976; Gerbner et al., 1986; Grabe and Drew, 2007; Bilandzic and Busselle, 2008; Lee and Niederdeppe, 2010; Morgan and Shanahan, 2010; Morgan et al., 2015). Consistent with this theorizing, the findings of the current study showed that the more US crime shows Austrians watched, the more they seemed to have contradicting “television facts” saved in their memory and tended to mistakenly believe that the death penalty is used in Austria. This current study adds important evidence to findings of previous studies suggesting that watching US crime shows predicts distorted perceptions of the domestic use of the death penalty in other countries (Truong, 2011; Till et al., 2016). More research exploring the mechanisms behind associations of television viewing with perceptions and beliefs related to the death penalty and other aspects of the justice system, particularly experimental approaches, are warranted.

## Data Availability Statement

The raw data supporting the conclusions of this article will be made available by the authors, without undue reservation.

## Ethics Statement

Ethical review and approval was not required for the study on human participants in accordance with the local legislation and institutional requirements. Written informed consent from the participants' legal guardian/next of kin was not required to participate in this study in accordance with the national legislation and the institutional requirements.

## Author Contributions

BT: conceived and designed the study, performed data collection, analyzed the data, and wrote the first draft of the manuscript. TN: supervision. TN and FA: provided important intellectual content in revising the manuscript. All authors contributed to the article and approved the submitted version.

## Conflict of Interest

The authors declare that the research was conducted in the absence of any commercial or financial relationships that could be construed as a potential conflict of interest.
