# Supplementary material for: The Relationship Between Crime-Related Television Viewing and Perceptions of the Death Penalty: Results of a Large Cross-Sectional Survey Study
Source: Front Psychol. 2021 Jul 21;12:715657. doi: 10.3389/fpsyg.2021.715657 (PMC8334551; doi:10.3389/fpsyg.2021.715657)
Supplement: Supplementary file 1 [file Data_Sheet_1.pdf]

Supplementary Table 1. *Unweighted Descriptive Statistics of Perception of the Death Penalty and Television Viewing among Study Participants (n = 1001)*

**Question**	**Answer**	***N***	**%**	**TV viewing**		**Watching US crime shows**	
				M	***SD***	***M***	***SD***
Anyone on death row	correct	866	86.5	29.01	20.35	7.33	11.31
	incorrect	135	13.5	31.59	24.41	14.65	19.96
Lethal injection past 5yr	correct	898	89.7	29.25	20.86	7.59	12.51
	incorrect	103	10.3	30.27	21.77	14.63	15.77
Lethal injection past 25yr	correct	857	85.6	29.22	20.36	7.55	11.46
	incorrect	144	14.4	30.19	24.21	12.86	19.50
Electric chair past 5yr	correct	919	91.8	29.27	20.76	7.61	12.50
	incorrect	82	8.2	30.30	23.08	16.28	16.18
Electric chair past 25yr	correct	885	88.4	29.38	20.91	7.65	12.57
	incorrect	116	11.6	29.21	21.32	13.42	15.36
All five questions combined[1]	correct	824	82.3	29.23	20.39	7.43	11.45
	incorrect	117	17.7	29.95	23.43	12.44	18.28

Values are weighted absolute (*N*) and relative (%) frequencies of participants answering the five questions on the death penalty correctly or incorrectly as well as their means (*M*) and standard deviations (*SD*) in terms of weekly television consumption and weekly consumption of US crime shows.

[1]This parameter was coded as correct, if all five questions on the death penalty were answered correctly, and as incorrect, if any of the five questions were answered incorrectly.

Supplementary Table 2. *Correlation Matrix: Unweighted Pearson Correlations between Perception of the Death Penalty, Television Viewing, Gender, Age, and Education*

	X1	X2	Y1	Y2	Y3	Y4	Z1	Z2	Z3	Z4	Z5	Z6	Z7
**Television viewing (X1)**	1.00												
**Watching US crime shows (X2)**	.41*** (.31, .51)	1.00											
**Gender[1] (Y1)**	.00 (-.06, .07)	-.06 (-.11, .00)	1.00										
**Age (Y2)**	.19*** (.14, .24)	-.03 (-.09, .02)	-.22*** (-.28, -.16)	1.00									
**Education: High school (Y3)**	-.06 (-.12, .00)	-.03 (-.10, .05)	-.05 (-.11, .01)	-.02 (-.10, .05)	1.00								
**Education: College (Y4)**	-.08* (-.13, -.02)	-.02 (-.07, .05)	-.06 (-.12, .01)	.05 (-.02, .11)	-.15*** (-.17, -.13)	1.00							
**Anyone on death row (Z1)**	.04 (-.03, .11)	.19*** (.12, .27)	.08* (.02, .14)	-.25*** (-.31, -.20)	-.01 (-.07, .05)	.04 (-.02, .11)	1.00						
**Lethal injection past 5yr (Z2)**	.02 (-.05, .08)	.16*** (.09, .25)	.07* (.01, .13)	-.26*** (-.31, -.21)	-.01 (-.07, .05)	.06 (-.01, .13)	.77*** (.71, .83)	1.00					
**Lethal injection past 25yr (Z3)**	.02 (-.05, .08)	.14*** (.07, .23)	.12*** (.06, .18)	-.31*** (-.37, -.26)	-.01 (-.07, .05)	.03 (-.04, .10)	.71*** (.64, .77)	.81*** (.75, .86)	1.00				
**Electric chair past 5yr (Z4)**	.01 (-.05, .08)	.18*** (.11, .27)	.05 (-.02, .11)	-.21*** (-.27, -.16)	-.02 (-.07, .05)	.07* (.002, .15)	.70*** (.63 .77)	.86*** (.81, .91)	.70*** (.64, .76)	1.00			
**Electric chair past 25yr (Z5)**	.00 (-.06, .06)	.14*** (.07, .23)	.11*** (.05, .17)	-.28*** (-.33, -.22)	.02 (-.04, .10)	.02 (-.04, 09)	.69*** (.62, .75)	.81*** (.75, .87)	.85*** (.80, .89)	.81*** (.76, .87)	1.00		
**All 5 questions combined[2] (Z6)**	.01 (-.06, .08)	.15*** (.07, .22)	.12*** (.05, .18)	-.33*** (-.39, -.28)	-.02 (-.07, .05)	.01 (-.05, .07)	.85*** (.81, .90)	.73*** (.68, .78)	.88*** (.85, .92)	.65*** (.58, .70)	.78*** (.73, .83)	1.00	
**Death penalty sum score (0-5)[3] (Z7)**	.02 (-.04, .09)	.18*** (.11, .26)	.10** (.04, .16)	-.29*** (-.35, -.24)	-.01 (-.06, .06)	.05 (-.02, .12)	.86*** (.83, .89)	.94*** (.92, .95)	.91*** (.88, .93)	.89*** (.86, .92)	.92*** (.90, .94)	.87*** (.85, .89)	1.00

Values are correlation coefficients (*r*) from unweighted Pearson correlations with bootstrapped 95% confidence intervals on 1000 samples given in parentheses; * $p < .05$, ** $p < .01$, *** $p < .001$ (two-tailed).

[1]Reference group: Male. [2]This parameter was coded as correct, if all five questions on the death penalty were answered correctly, and as incorrect, if any of the five questions were answered incorrectly. [3]This parameter is a sum score based on the responses to the five death penalty questions (0 = correct answer, 1 = incorrect answer) ranging from 0 to 5.

Supplementary Table 3. *Results of Binary Logistic Regression Analyses to Predict Perceptions of the Death Penalty among Study Participants*

**Question**		**TV viewing**	**Watching US crime shows**	**Gender[1]**	**Age**	**Education: High school**	**Education: College**
Anyone on death row	*b* (SE)	0.00 (0.01)	0.04 (0.01)	0.26 (0.21)	-0.51 (0.07)	-0.17 (0.30)	0.46 (0.29)
$\chi^2(6) = 103.37$, $p < .001$	Wald	0.11	22.71***	1.60	52.71***	0.32	2.43
Nagelkerkes $R^2 = .18$	OR	1.002	1.036	1.295	0.601	0.844	1.577
	(95% CI)	(0.991 – 1.012)	(1.021 – 1.052)	(0.867 – 1.934)	(0.524 – 0.690)	(0.469 – 1.518)	(0.890 – 2.797)
Lethal injection past 5yr	*b* (SE)	0.00 (0.01)	0.04 (0.01)	0.24 (0.23)	-0.64 (0.09)	-0.33 (0.35)	0.68 (0.31)
$\chi^2(6) = 105.39$, $p < .001$,	Wald	0.08	17.84***	1.09	54.49***	0.90	4.73*
Nagelkerkes $R^2 = .21$	OR	0.998	1.036	1.274	0.527	0.720	1.975
	(95% CI)	(0.986 – 1.010)	(1.019 – 1.053)	(0.809 – 2.007)	(0.445 – 0.625)	(0.366 – 1.419)	(1.070 – 3.644)
Lethal injection past 25yr	*b* (SE)	0.00 (0.01)	0.03 (0.01)	0.49 (0.21)	-0.64 (0.07)	-0.31 (0.30)	0.37 (0.30)
$\chi^2(6) = 134.39$, $p < .001$,	Wald	0.17	13.86***	5.59*	74.40***	1.11	1.59
Nagelkerkes $R^2 = .22$	OR	1.002	1.029	1.627	0.527	0.731	1.454
	(95% CI)	(0.992 – 1.013)	(1.013 – 1.044)	(1.087 – 2.435)	(0.455 – 0.609)	(0.408 – 1.309)	(0.813 – 2.600)
Electric chair past 5yr	*b* (SE)	-0.01 (0.01)	0.04 (0.01)	0.18 (0.25)	-0.54 (0.09)	-0.30 (0.39)	0.80 (0.33)
$\chi^2(6) = 80.77$, $p < .001$,	Wald	0.83	22.09***	0.48	35.83***	0.61	5.95*
Nagelkerkes $R^2 = .18$	OR	0.994	1.042	1.191	0.580	0.738	2.222
	(95% CI)	(0.980 – 1.007)	(1.024 – 1.060)	(0.726 – 1.954)	(0.486 – 0.694)	(0.346 – 1.577)	(1.170 – 4.220)
Electric chair past 25yr	*b* (SE)	0.00 (0.01)	0.03 (0.01)	0.51 (0.23)	-0.60 (0.08)	0.03 (0.30)	0.38 (0.32)
$\chi^2(6) = 108.11$, $p < .001$,	Wald	0.29	15.91***	5.15*	55.34***	0.01	1.41
Nagelkerkes $R^2 = .20$	OR	0.997	1.032	1.665	0.550	1.029	1.461
	(95% CI)	(0.985 – 1.009)	(1.016 – 1.049)	(1.072 – 2.585)	(0.470 – 0.644)	(0.571 – 1.854)	(0.781 – 2.733)
All five questions combined[2]	*b* (SE)	0.00 (0.01)	0.03 (0.01)	0.38 (0.19)	-0.62 (0.07)	-0.34 (0.28)	0.20 (0.28)
$\chi^2(6) = 148.48$, $p < .001$,	Wald	0.18	15.02***	4.00*	86.95***	1.50	0.48
Nagelkerkes $R^2 = .23$	OR	1.002	1.028	1.456	0.537	0.713	1.217
	(95% CI)	(0.992 – 1.012)	(1.014 – 1.043)	(1.008 – 2.104)	(0.471 – 0.612)	(0.416 – 1.224)	(0.698 – 2.122)

Values are unweighted unstandardized regression coefficients (*b*) with standard errors (*SE*) given in parentheses, Wald statistics, and odds ratios (OR) with 95% confidence intervals (95% CI) given in parentheses; * $p < .05$, ** $p < .01$, *** $p < .001$ (two-tailed).

[1]Reference group: Male. [2]This parameter was coded as correct, if all five questions on the death penalty were answered correctly, and as incorrect, if any of the five questions were answered incorrectly.

Supplementary Table 4. *Results of Multiple Linear Regression Analysis to Predict Perceptions of the Death Penalty[1] among Study Participants*

**Predictor**	R^2	ΔF	***B***	**SE *B***	**β**	***t***
	.12	23.04***				
TV viewing			.00	.00	.01	0.21
Watching US crime shows			.02	.00	.17	5.24***
Gender[2]			.14	.09	.05	1.56
Age			-.23	.03	-.28	-8.97***
Education: High school			.02	.13	.01	0.17
Education: College			.31	.14	.07	2.25*

Values are unstandardized (*B*) und standardized (β) regression coefficients, standard errors of the unstandardized regression coefficients (SE *B*), and *t* values (*t*) based on unweighted data. Also reported are R^2 and change in *F* value (ΔF) of the regression model; *p<0.05, **p<0.01, ***p<0.001 (two-tailed).

[1]This parameter is a sum score based on the responses to the five death penalty questions (0 = correct answer, 1 = incorrect answer) ranging from 0 to 5. [2]Reference group: Male.
